# Dropout analysis: A method for data from Internet-based research and dropR, an R-based web app and package to analyze and visualize dropout

**DOI:** 10.3758/s13428-025-02730-2

**Published:** 2025-07-18

**Authors:** Ulf-Dietrich Reips, Annika T. Overlander, Matthias Bannert

**Affiliations:** 1 Department of Psychology, University of Konstanz, Universitätsstr. 10, 78464 Konstanz, Germany; 2 ETH Zurich, Zurich, Switzerland

**Keywords:** Dropout, Atrittion, Mortality, Non-response, Web app, R package, Shiny, iScience, Survival analysis

## Abstract

With Internet-based research, non-response such as lack of responses to particular items and dropout have become interesting dependent variables due to highly voluntary participation and large numbers of participants (Reips, 2000, 2002b). In this article, we develop and discuss the methodology of using and analyzing dropout in Internet-based research, and we present dropR, an R package and web service (web application) to analyze and visualize dropout. The web app was written in R using Shiny, a free software environment for statistical computing and graphics. Among other features, dropR turns input from datasets into accessible and publication-ready visual displays of dropout curves. It calculates parameters relevant to dropout analysis, such as chi-square values and odds ratios for points of difference, initial drop, and percent remaining in stable states. It provides Kaplan–Meier survival statistics and tests survival curve differences. With automated inferential components, it identifies critical points in dropout and critical differences between dropout curves for different experimental conditions (Kolmogorov–Smirnov and rho-family statistics) and produces related statistical copy. Requiring no programming knowledge, dropR is provided as a free web application at https://dropr.eu and for programmers as an R package (under a cost free general public license, GPL-3, https://cran.r-project.org/web/licenses/GPL-3) from researchers for researchers. All code and materials are openly available on GitHub (https://github.com/iscience-kn/dropR).

## Introduction

Computer-based data collection and Internet-based data collection, with its widespread use in particular, have offered routine access to measures that were impossible or difficult to achieve in traditional behavioral and self-report studies. These measures include *paradata* (e.g., information about changes in responses, mouse movements, Heerwegh, 2003; Stieger & Reips, 2010), *metadata* (e.g., information about technologies used; Reips, 2009; Schmidt, 2007), and *dropout*.

**Fig. 1:**
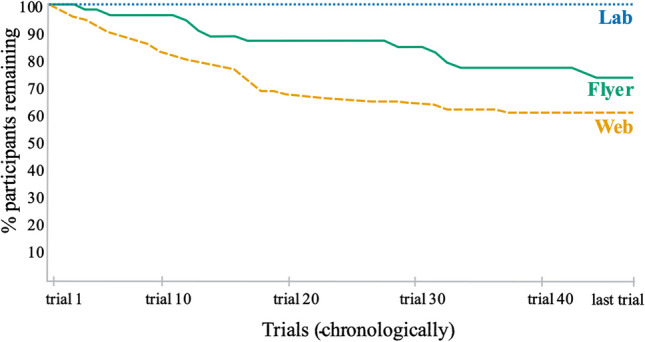
Dropout rates by the method of recruitment. *Note*. Dropout rates resulting from an experimental manipulation of recruitment of participants and setting: No dropout in the laboratory, after traditional recruitment, but on the Internet, both when recruited via flyer or via links on websites (figure adapted from Reips, 2002c, based on data from Neuhaus, 2002).

Dropout is generally much more likely in Internet-based than laboratory-based research because of higher “voluntariness” on the Internet, i.e., participants are usually free to drop out throughout the study (Musch & Reips, 2000; Reips, 2000, 2021). Lack of personal face-to-face contact with an experimenter and subjectively felt social “pressure” to remain in the study situation likely contribute to this difference between Internet-based and laboratory-based research (Reips, 2002b). Internet-based participants can more easily get distracted, as the lure of many apps and alternative attractors on their devices is less controlled than in the laboratory, where the setting and computer setup are designed, and experimenters are determined to keep participants in focus. In a signal-detection experiment conducted with Internet technologies as part of a master’s thesis (Neuhaus, 2002), we had participants either take part in the lab or on the web. Web participants were further recruited either via a flyer handed to them on campus that contained the study’s URL or found the study on the web. Figure 1 shows the dropout curves for the three resulting conditions, and dropout in later studies generally tended to be in line with these results (Reips, 2021). However, this difference in settings and recruitment can be overcome by motivationally relevant procedures: Frick et al. (2001) showed that dropout in Internet-based research can be dramatically reduced by early self-commitment to the study and expectations about financial incentives. Galesic (2006) found that announced length of study, respondents’ age, interest, and burden to significantly affect the risk of dropout. Further reasons for a higher incidence of dropout in Internet-based research lie in technology and interactions between user factors and technology. For example, Schwarz and Reips (2001) showed that, at the time, the use of client-side JavaScript caused more dropout than server-side scripting. Stieger et al. (2011) similarly found detrimental effects of using Java applets (a different technology than JavaScript) in web-based studies on dropout and sample composition.

Because dropout is more frequent in Internet-based research, three solutions were proposed as possible ways of action for researchers who wish to conduct Internet-based research (Reips, 2002a, 2007): Avoiding dropout, controlling dropout, and using dropout.

*Avoiding dropout* means using techniques that prevent dropout-inducing factors from playing out during a study. Examples include the high hurdle and warm-up techniques (e.g., Reips, 2000) and building studies in ways that follow the low-tech principle, i.e., not using technologies that are too difficult for users or have a high likelihood of technological incompatibilities.

By *controlling dropout,* one asks and records participants’ intentions to drop out or not. The so-called seriousness check (Aust et al., 2012; Reips, 2000, 2009) has been shown to predict dropout effectively and thus can be used to exclude data sets by participants who intend to drop out or not take participation seriously. Reips (2009) observed that the seriousness check is the “… single best predictor for dropout. Of those answering “I would like to look at the pages only” around 75% will drop, while of those answering “I would like to seriously participate now” only ca. 10–15% will drop. Overall, about 30–50% of visitors will fail the seriousness check, i.e., answer “I would like to look at the pages only.” (p. 54).

*Using dropout* means turning dropout into a dependent or a diagnostic variable. Observing frequent dropouts at certain items during pretesting of questionnaires allows the researcher to diagnose items that may be difficult to understand, sensitive, or impossible to answer. The substantially larger incidence of dropout in Internet-based versus laboratory-based research generates enough variance to study the effects of experimental manipulations on dropout. Examples by Frick et al. (2001) and Knapp and Heidingsfelder (2001) demonstrate how dropout can be used as a natural dependent variable in research on Web survey methodology.

A fourth option, suppressing non-response, including dropout, reduces voluntary participation to the point of forcing participants to complete the study and has been shown to create psychological reactance that leads to low data quality (Stieger et al., 2007).

Reips (2002a) recommends using dropout as a dependent variable and describes how it may be used for detecting *motivational confounding* by looking at dropout curves by experimental conditions. If there is a higher dropout in one condition and no technology confounds or other obvious alternative reasons are present, then it can be concluded that the condition is more boring or otherwise less attractive to the participants. Comparisons of causal effects by experimental conditions on other dependent variables would therefore be confounded. Such confounding cannot easily be detected in lab research, where there is usually no dropout. Lithopoulos et al. (2020) and Sheinbaum et al. (2013) have been following this line of thinking about the advantageous web mode due to motivational confounding and added to its empirical foundation.

Researchers should be aware that even with similar dropout rates in conditions, they may end up drawing wrong conclusions about the direction of an experimental causal effect if participants drop out of different conditions for different reasons. Birnbaum and Mellers (1989) describe how this could happen and recommend checking demographic or other background variables of dropouts for any correlations with the experimental conditions. Therefore, it is recommended to assess background variables before introducing experimental conditions.

## Measuring and reporting dropout

Dropout is most often reported as percentages; differences in dropout are usually reported as results from chi-square tests, Fisher’s exact tests, survival analyses, or odds ratios. We believe the most intuitive and informative way is to report dropout as *dropout curves by condition* (see examples in Fig. 1). Reviewers of manuscripts that report research involving Internet-based experiments should always request dropout curves so that effects of and possible confounding via differential dropout can be detected.

Because it has been known for some time that dropout can be used as a dependent measure in Internet-based research, several other tools have features that prepare data sets to ease later dropout analysis. WEXTOR (https://wextor.eu/, Reips & Neuhaus, 2002) saves a “1” to the data file for each page that was visited by a participant, so dropout becomes visible in a standard spreadsheet – this is especially valuable in a so-called one-item-one-screen design (OIOS), where each webpage corresponds to a single item (Reips, 2021). Scientific LogAnalyzer (Reips & Stieger, 2004) creates a dropout tree that allows the researcher to visualize for each page the branching of participants into further participation versus dropout.

Furthermore, we are aware of three other current R packages that seek to analyze dropout (or attrition): The “dropout” package (Mann, 2024) simply offers one function for detecting dropout and another for summarizing dropout statistics for each column in the original data. This package is available on both GitHub and CRAN.

Two other available packages, “attritevis” (Lo et al., 2024) and “attrition” (Coppock, 2022) serve distinct purposes. Generally, *attritevis* focuses on the visualization of attrition (as the name suggests), while the *attrition* package is more focused on value-bound estimation in the context of missing data. The dropR package includes both important aspects in one package with clearly named functions, such as plot_do_curve() for an empirical overview of dropout over time and plot_do_kpm() for a visualization of the Kaplan–Meier survival statistics, as will be explained later in this article. Even though the *attritevis* package also provides some of the statistical analyses provided by the *attrition* package, survival analysis is lacking. The aforementioned packages are only available as developer versions from GitHub as of writing this, while dropR is officially available on CRAN. Additionally, finding the documentation for dropR is easy on both CRAN as well as the package vignettes available at https://iscience-kn.github.io/dropR/. We have put a strong focus on writing comprehensive documentation for the package and all its functions, as well as walk-through examples. Furthermore, we have put great efforts into making dropR as user-friendly as possible.

Most notably, dropR is the only dropout-focused software available that is entirely available as a web app that can be used for dropout analysis via a web browser without any programming knowledge. Like the R package, the dropR web app includes extensive instructions on how to use the tool properly to ensure the best analysis results. dropR thus offers a beginner-friendly environment that can also be used by users with more advanced programming knowledge.

## Three options of using dropR: Via web, in R or on one’s own server

The simplest way of using dropR is going to its website, https://dropr.eu (alternatively https://iscience-kn.shinyapps.io/dropR/), and using its browser interface. Of course, to use this option, you will need to be connected to the Internet throughout the analysis. If you choose this option of using dropR, then follow the *first* step-by-step guide, further marked with *Option 1* below, to conduct a dropout analysis. We recommend reading this section first as it provides a conceptual overview of how dropout analysis should be done in general (using the dropR web app to illustrate each step).

However, readers familiar with *R* may choose the second option and make use of dropR as an R package. The *second* step-by-step guide below presumes you have already downloaded and installed the *R* environment suitable for your computer (see https://cran.rstudio.org/). R is a freely available programming language and environment for statistical computing and graphics, which provides a wide variety of statistical and graphical techniques (R Core Team, 2024). Note that there are new versions of R available at least annually, and we recommend using the latest version (R 4.5.0 as of the writing of this manuscript in April 2025). Among the packages available are many for statistical tests, linear and nonlinear modeling, time series analysis, classification, clustering, and so on. The packages are all open source and thus can be modified by anyone if the original license is applied to the new versions.

The third option is to use the dropR web app and host it on one's own server. As this is the most specific use case, we will explain it last. However, for users with limited R experience and a high need for data privacy (e.g., due to sensitive research data or local regulations), this may be the best-suited option. Readers who identify with this user description may like to read the section on Option 1 and then continue reading the section on Option 3.

The remainder of this article is organized as follows: We will first describe a step-by-step dropout analysis via https://dropr.eu. This can be done with a web browser via an Internet connection; no programming knowledge is needed (*Option 1*). Then, we will describe how to install the dropR package on your local machine and walk you through a dropout analysis done locally in R (Option 2). Then, we’ll describe how to install dropR on a web server that you can run for a class or group locally (Option 3). The advantages of and options for the different routes are discussed. Finally, we’ll report on results from systematic tests with different operating systems and browsers.

## Option 1: Conducting dropout analysis with the dropR web app, step-by-step

**Fig. 2:**
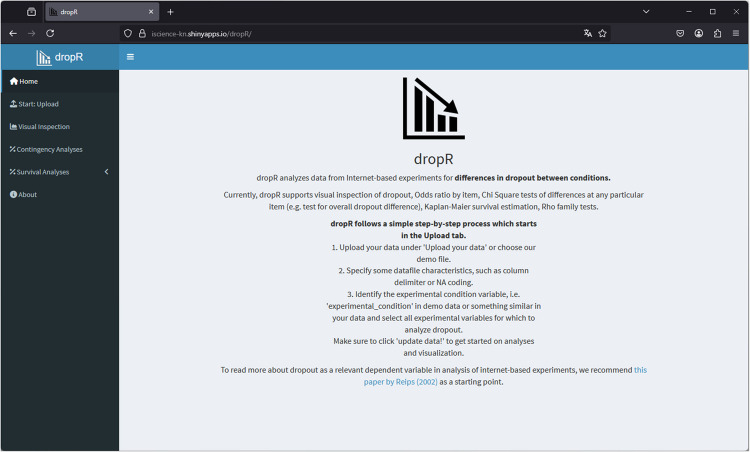
Initial page shown in the dropR application

A main contribution of *dropR* is its web-based graphical user interface (GUI) shown in Fig. 2. Using the Shiny web application framework (Chang et al., 2024), *dropR* provides a web interface to conveniently analyze dropout in an experiment or survey dataset. Thanks to the tool’s web GUI, the dropout analysis can be conducted without R or other programming knowledge using a standard web browser. Within a web browser dropR works with both mouse/key and touch interfaces. Even frequent R users who may want to follow our step-by-step guide for R savvy users (*Step-by-step example for R savvy users*, see Option 2 below) can profit from using dropR via the web app at https://dropr.eu or on a separate server, e.g., for teaching or running analyses on a mobile device such as a tablet or smartphone.

**Fig. 3:**
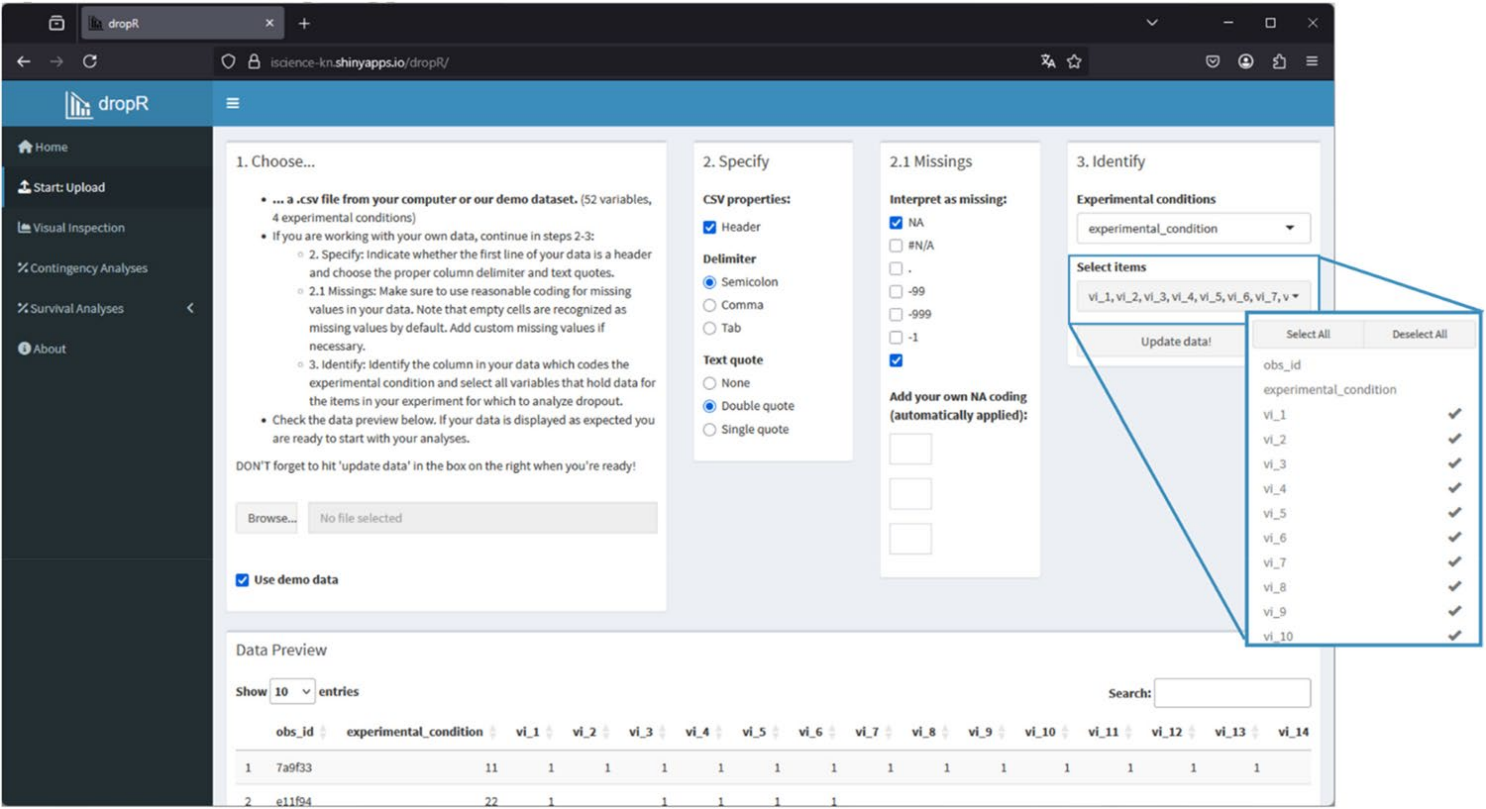
Upload tab in the dropR application

In *Step 1*, the survey or experiment data file should be saved as a comma-separated values (CSV) file. We made CSV the default format because CSV is widely used, and much software for Internet-based research (e.g., WEXTOR) and data analysis (e.g., R, Excel, SPSS) knows this format. It is crucial that the data are ordered chronologically as in the experiment or survey itself, because dropout is identified as sequences of missing data starting at the end of individuals' data in the dataset. As later the user needs to identify only the items containing experimental questions plus the item coding experimental condition, it may be wise to create a dataset upfront that only contains those items in the correct order. In any case, it is advisable to have the variable identifying experimental conditions at the very front of the dataset for ease of use and clarity. Within the dropR app, you upload the data file in the “Start: Upload” tab, see Fig. 3 upper left. If the data file is in another format than CSV, different separators (i.e., semicolon, tab) can be chosen as well as types of identifying quotes. When users upload data files to the dropR web app (hosted on shinyapps.io), the data are stored within the application’s private file system. This storage is ephemeral, meaning any files modified during a session will persist only while the application is running. Once the application idles and transitions to a sleeping state, or if the application is restarted, any changes to the file system – including uploaded data – are lost. This ensures that sensitive research data can safely be uploaded for analysis with dropR and will neither be available to the developers nor any other user. It will also not be available to the same user between sessions, so this is a feature to keep in mind.

*Step 2*: Choose the proper coding of missings adequate for the dataset. There are many options of coding for missings provided in dropR, such as nothing, “NA” or “N/A”. We further provide three fields for custom coding of missings, to reduce the need for data preprocessing before upload. The data preview at the bottom will update live and show the resulting data from the choices made to ensure the correct format.

Importantly, dropR is able to identify dropout while being immune to single or multiple points of missings within the data. It happens that participants may choose to skip an item because they do not want or are not able to give an answer. Another scenario is that participants simply forget to fill in some items and are not alerted of this in the experiment (or they are and still want to move on, which is called “soft validation”). Generating the dropout index in dropR is immune to such behaviors and only counts missing data as dropout if there are no more responses until the end.

*Step 3*: In the right part of the Upload page, as shown in Fig. 3, choose one column that identifies the experimental or quasi-experimental conditions and under “Select items” all columns that hold the item or progression variables. These variables need to be listed in the sequence they were presented during the study to ensure meaningful analyses. If variable names were created in an experiment generator (e.g., in WEXTOR) this will most likely be the case. Hit the “Update data!” button.

**Fig. 4:**
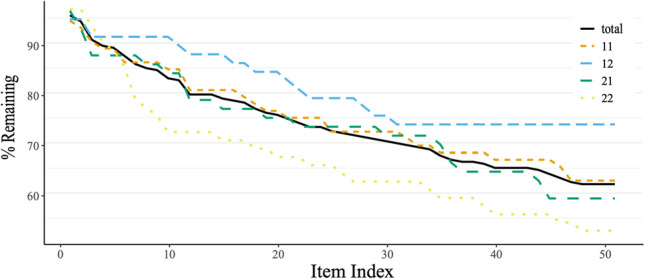
Example of dropout analysis for four experimental conditions. *Note*. The dropout curves shown here were created using the default settings in the dropR web app. The data used is the demo dataset with fictitious data available in the package as dropR::demodata or in the app by checking the “Use demo data” box in the Upload tab. It shows dropout rates for four experimental conditions (11, 12, 21, 22) over 52 questions as well as the overall dropout curve (“total”)

*Step 4*: Moving on to the “Visual Inspection” tab will generate the dropout analysis based on the items selected in Step 3, with visualization depicted in Fig. 4. Note that for *x* selected items, *x-1* dropout steps will be displayed by default in dropR visualization, because the number of participants at the first selected item is taken as 100%, from which all other selected items mark relative drop. This policy allows for analyses of dropout beginning at any point during an experiment, e.g., comparative dropout after an intermediate instruction. We will now explain several visualization and analysis features of dropR with the help of Fig. 4.

The main part of the panel displays dropout curves by condition and in total. Display of each of the curves can be turned off or on by checking the corresponding box on the left under “Selected conditions”. Percent of participants remaining is depicted on the vertical axis, and the succession of items or pages on the horizontal axis.

*Step 5:* *Detecting dropout points and differences between conditions*. By visual inspection, likely candidates for differences in dropout can be identified on the curves. Curves are displayed with different line types (dashes, points, combinations of both, solid) and adjustable stroke widths for optimal visualization. To further optimize the information carried in the visualization one can choose from different color palettes (color-blind friendly, ggplot2 default, grayscale) to display the curves. The analyses sections (Contingency and Survival) provide statistical measures for comparing dropout between conditions. In the Contingency section, the chi-square table will change according to the slider on the left, with which you select the question to compare dropout rates at and display statistics for the selection. This feature can be used to test a specific hypothesis for a dropout difference at a particular point of the study, or it can be used exploratorily, e.g., to detect items in a questionnaire that trigger dropout. The odds ratio condition matrix at the bottom of the window displays calculated values for all combinations of conditions. Chi-square and odds ratio statistics are the most commonly used ways of reporting differences in dropout analyses.

*Step 6:* *Statistical analyses*. Initially, the output will show statistics for the overall dropout, i.e., for differences between conditions in reaching the end of the study. Currently, dropR calculates chi-square tests and odds ratios (located in the Contingency Analyses section in the app), Kaplan–Meier statistics, rho-family survival differences, and a Kolmogorov–Smirnov test (located under the Survival Analyses menu in the app) for calculating dropout difference between the most extreme conditions.

Under the menu “Survival Analyses,” there are the survival test statistics as well as survival curve plots that can be downloaded individually. The survival statistics are based in large part on the *survival* package (Therneau, 2024). Moreover, under “Explanation” there is further in-depth information on survival analysis, especially in the context of dropout. As these types of analyses and methodologies of web-based research are not yet commonly taught in curricula (e.g., Krantz & Reips, 2017), we provide these resources within dropR.

*Step 7: Visualization*. A feature of dropR is that all visual displays and analyses are instantly updated with changes in input. The two sections, “Home” and “About,” display additional information on dropout in general, how to use dropR, and who its authors are. Figures of dropout curves can be configured regarding accessibility, resolution, width, and height, and they can be downloaded from the app. Color-blind-friendly palettes support up to eight different conditions (colors). Note that several common file formats are available, such as PDF and PNG, and vector graphics in SVG format. We recommend using the latter type of image to ensure the best possible quality of figures, as it is scalable without depending on pixels for display.

## Option 2: Using dropR in R – Getting started with the package and local app

Setting up dropR is easy and can be done interactively from an R session running R version 3.0.0 or higher. Installing the appropriate version of R for your computer and operating system can be done by following the instructions on https://cran.rstudio.com/. R is a fairly lightweight installation, so it should run on most computers, although the Shiny package can require up to 50 MB of RAM. To start the initial download and installation, simply evoke R and run the following line of code in your R console. Alternatively, if you use R Studio (Posit Team, 2024) as an Integrated Development Environment or editor you might want to use the program’s GUI and select the package from the packages pane.
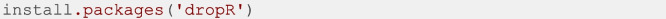


Once the package and all of its dependencies are resolved, the installed package can be loaded into the R session. As always in R, beginning to work with the package is done by typing “library(dropR)” into the R console or script every time a new R session is started to load the package. If dropR functions are included in a script, then dropR should be loaded at the beginning of the script.

Then, once dropR is installed locally and loaded, there are two main ways of making full use of the package:

First, it can be used via the Shiny application and started from inside an R session by typing “start_app()” into the R console. It will open the Shiny app locally with an easy-to-use interface as well as guided instructions on how to conduct the dropout analyses. R will remain active in the background and use its own built-in web server to serve the content. This way of using dropR is helpful for experienced users but allows inexperienced users to conduct analyses directly in R as well. Figure 2 shows the dropR Shiny app running on the local built-in web server that is preinstalled on most modern operating systems. The web-based version of dropR at https://dropr.eu is being run online using the same Shiny application as the one that can be used locally.

Second, dropR can be used as a regular R package, independent from the Shiny app. All functionalities implemented in the app are split into their own R functions, which are made available to users when installing and loading the package. The dropR package provides several functions that can be used to write R scripts or reports in RMarkdown (Allaire et al., 2024). A thorough documentation of each function, as well as example usage and a walk-through guide, can be found at https://iscience-kn.github.io/dropR. As with any other R package, help pages are also available directly in R. For example, typing “?dropR::add_dropout_idx” into the console will show help to users on how to get started with dropR by adding the index of dropout to their own data or the demo dataset called “dropRdemo”, which is included in the package installation.

## Step-by-step example for R-savvy users

Readers who are advanced users of R may want to use dropR beyond the web application, i.e., within their interactive R environment, and customize dropR functionality during their analyses. We have released the current R package version of *dropR* to the official R repository, the Comprehensive R Archive Network (CRAN), where it was recently accepted (Overlander et al., 2024; as was the earlier version at the time, Reips & Bannert, 2014, 2015). Moreover, the latest development version of dropR can be installed from the GitHub repository. We recommend doing so directly in R, for example using devtools::install_github(“iscience-kn/dropR”). The official dropR version can be installed directly from CRAN by using install.packages(“dropR”) in R.

The following walk-through of dropR can also be found in the package vignettes online (see how-to-guide at https://iscience-kn.github.io/dropR/articles/interactive.html). In a step-by-step fashion, we describe demo analyses using the provided dataset “dropRdemo” from the package.

*Step 1* is to begin by loading the package as shown in the code chunk below. We recommend copying the data frame into a new one for more clarity and to be able to easily change or redo any analyses or edits; therefore we will copy the data to the data frame. As a *second* *step*, the dropout index will be added for each person, i.e., the number of the last question answered (or webpage visited) is to be added as a new column to the data frame. We will hard-code the number of questions in the data that contain the questions, but depending on the variable names, it may be useful to soft-code this. The added dropout index ‘do_idx’ column is the main basis for analyses in the dropR package, so do not skip this step.

The *third* *preparatory step* will be to create a new data frame that contains dropout statistics by experimental condition and serves as an overview as well as a basis for overall visualization. On the pre-modified data frame ‘df’, which now contains a column called ‘do_idx’ for dropout index, we will run the ‘compute_stats()’ function and define the column containing information on the experimental condition and the number of variables that code questions:
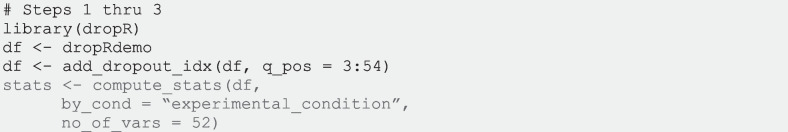


The new data frame ‘stats’ contains columns with information on the question order, the condition, the cumulative sum of dropout (‘cs’), the sample size at the beginning, the remaining participants in absolute numbers, and percent remaining. If no experimental condition is chosen inside the function, this table will only contain information on the overall condition; if it is chosen as shown above, the table also contains the information grouped by condition.

*Step 4*: After data preparation, all other functions of dropR can be run. To plot the data as-is, simply call plot_do_curve(stats). This function is highly customizable, e.g., by differentiating line types depending on the experimental condition (‘linetypes = TRUE), “zooming in” on the relevant data by scaling down the *y*-axis (full_scale = FALSE) or showing confidence bands in the plot (show_confbands = TRUE). We invite users to consult each function’s help page for more information, either directly in R, e.g., by typing?plot_do_curve inside the console, or by reading the function-specific vignettes online. It is also possible to do all data preparation directly inside the plot function. This can be useful for a quick overview of the data if no other functions are to be used with the prepared data. For this, we can call:
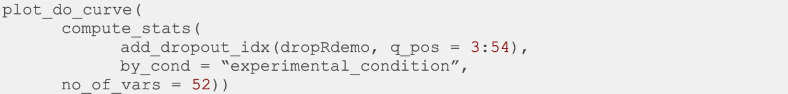


*Step 5*: dropR offers many functions for statistical analyses of dropout. Some need the previously created data frame here called ‘stats’, some take the original data with added column ‘do_idx’ as input, so please make sure to input the right type of data for each function. As explained in Step 2, the ‘do_idx’ serves as the basis for calculation, e.g., Kaplan–Meier needs your original data with the added dropout index (from function add_dropout_idx) while Kolmogorov–Smirnov needs the dropout statistics as input (from function compute_stats, which in turn relies on the do_idx). Please consult the help files to see, which type of data is required as input for which specific function, e.g., with ?dropR::do_ks in the R console for help on how to calculate Kolmogorov–Smirnov. You can also find an overview of explanations for each function online at https://iscience-kn.github.io/dropR/reference/.

An easy way to visualize survival data with dropR is to use nested functions. It is, of course, possible to create intermediate objects and use those, but sometimes it might be more efficient to define everything inside one function call. To create a Kaplan–Meier plot, the function needs a survival object as input. Keep in mind that the do_kpm() function needs the df, which already has the do_idx added as input. Thus, we can call: 
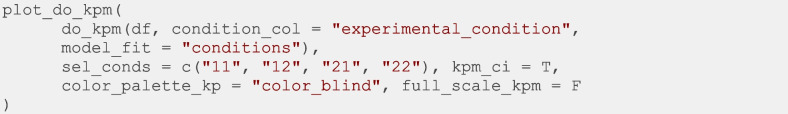


*Step 6*: For an even more condensed analysis and visualization, the Kolmogorov–Smirnov (KS) analysis compares dropout rates from the most different conditions by question, i.e., the conditions with the least and most participants remaining (in percent relative to the sample size in the respective experimental condition). We will create a KS dropout plot of the previously created prepared data at the last question, in our demo case, that is question 52:
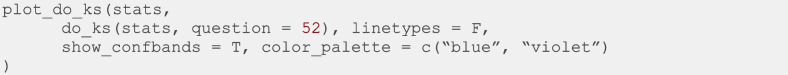


Notice that this plotting function allows the user to define custom colors for the visualization. Since all plotting functions in dropR are based on ggplot2, keen users may want to customize the outputs even further, for example, by adding custom labels with ggplot2::labs().

## Option 3: Installing and using dropR as a web service

The web service^1^ at https://dropr.eu only requires an Internet connection to use dropR; no installation of software is needed. As an alternative to using this web service or running a local web server on the same machine, dropR can be installed on a Shiny web application server. Such a web server makes the dropR application described above available as a web service to online users without requiring them to download R and install the dropR package. For advantages of web services and web applications, see Reips and Lengler (2005). Installing your own Shiny web application server is the way to go if you would like to provide your own controlled version and space for dropR, e.g., to handle confidential data within your own research group or provide the service in-house for teaching groups of students. The Shiny server is available as a licensed, cost-free, open-source community version as well as in an Enterprise grade version (Posit Connect). Keep in mind that the provided Shiny app and dropR are useful in teaching students the importance of dropout analysis.

To install your own dropR Shiny app, download the package source from CRAN to your local machine, i.e., your own web server, or navigate directly to the “*inst*” folder on the dropR GitHub (https://github.com/iscience-kn/dropR/tree/main/inst). Untar (i.e., decompress) the package archive and browse to the resulting folder. In both cases, go to the *inst* folder and copy the entire *app* folder to the web server’s document folder (e.g., *public*). This will make the *dropR* app available at https://yourserver.edu/public/app, where "yourserver.edu" needs to be replaced by your own server's domain. We have registered the official dropR Shiny app at https://dropr.eu where it has been running freely and openly with worldwide accessibility since February 1, 2015 (Reips & Bannert, 2015).

## Empirical tests

Dropout is sometimes caused by technological issues, as participants in Internet-based research use a multitude of different devices with different operating systems and web browsers. Measurement that depends on such variations in technology may lead to varying results (Garaizar & Reips, 2019; KuhlMann et al., 2021; Warburton et al., 2025). Thus, any thorough dropout analysis should include comparisons of dropout rates by type of device and software used. As our software also relies on browsers and operating systems (depending on the chosen type of use described above) we ran a number of empirical tests on dropR that we will describe below.

To assess the performance and cross-platform compatibility of the dropR Shiny application, we conducted a series of system tests focused on *file upload* and *processing speed*. A test file of approximately 2 MB was used to simulate a large data upload scenario (in the context of typical data file sizes in the experimental behavioral sciences). It contained arbitrary data for 1000 participants in 100 variables and two conditions. This corresponds to the dimensions of an extensive online experiment data set with a large sample size. The tests were carried out on both Windows and Mac operating systems, utilizing four commonly used browsers: Firefox, Safari, Edge, and Chrome. Across all environments, the application consistently completed file uploads and initial processing within a time range of 1–2 s, showing minimal variance of solid performance between browsers or operating systems. This also held true for a mobile test carried out on an Android smartphone (One Plus 9 Pro) using the Chrome browser. The longest processing times for any of the procedures in the dropR app were recorded for Kolmogorov–Smirnov survival analysis due to a need for more demanding pre-processing of the data. Even here, the expected loading time with a reliable Internet connection did not exceed 2 s.

These results indicate that dropR is well optimized for handling data uploads efficiently across diverse platforms. The consistent performance suggests that the web app is capable of managing files of varying sizes in typical user environments, including those on mobile devices. This broad compatibility and responsiveness make dropR a robust and user-friendly solution for data handling in laptop, desktop, and mobile contexts.

## Summary and outlook

Dropout analysis has become important because of the higher frequency with which substantial dropout is observed with the proliferation of Internet-based research. In the present article, we develop and discuss the methodology of avoiding, controlling, using, visualizing, and analyzing dropout, and we introduce dropR as an R package and web service for the purpose of dropout analysis.

The analysis of dropout in Internet-based experimenting is of particular importance because the prevalence of high dropout rates allows for the detection of motivational confounding in experiments (e.g., Reips, 2021). Furthermore, we would like to point out that while dropR is specifically designed to compare dropout between experimental conditions, it can, of course, also be used to compare any other groupings of interest. Depending on the research question, it could be interesting to compare dropout between categories in demographic variables such as gender, age group, or education or in psychological variables such as perception of anonymity e.g., between individuals who perceive their anonymity to be very important versus not important (Joinson et al., 2006). dropR expects input of a variable that codes experimental conditions, but users may input any variable that distinguishes a reasonable number of conditions (up to eight will be supported in the web app). These variables can be used as the grouping variable instead of the experimental condition, allowing analyses with a whole different aim. As in Fig. 1, it may often be wise to also compare dropout rates for different ways of recruitment of participants or sampling.

We strive to keep implementing new features in future versions of dropR, and because both R and dropR are open source, we hope for further development from members of the scientific community. While others have begun to recognize the issue of dropout handling, such as with the ‘dropout’ package in R (Mann, 2024), we believe dropR offers more usability for experienced as well as inexperienced users. Not only can it be used as both a package in R and via https://dropr.eu, in contrast to the ‘dropout’ package dropR allows for statistical analyses, such as chi-square, Kolmogorov–Smirnov, odds ratio, Kaplan–Meier, and rho family tests with instructions, visualizations, and publication-ready plotting options.

The large number of participants and highly voluntary participation in Internet-based research have enabled researchers to study the drivers of non-response (see e.g., Galesic, 2006; Reips, 2000), and several possible strategies to deal with dropout have been developed (Reips, 2012). dropR will be a useful tool in such research, and we hope it will be of use, for example, in web survey methodology and usability studies.

One of our motivations for creating dropR in the open-source R language is the framework’s remarkable success and the appeal of open-source in general. R is frequently used by empirical researchers of all disciplines, and its extension package repositories have established themselves as important parts of scientific computing.

Finally, with dropR, we hope to serve and reach those empirical scholars who have no or only minor programming skills for statistical analysis and visualisation, and we also hope it will appeal to those who like to use a programming language for these tasks. dropR makes use of recent advances in R and Shiny programming that provide an easy-to-use web interface on the one hand and on the other hand also allow one to look at its functions and use them in a scripting-based analysis. The dropR web app version intends to provide access for the large number of empirical researchers who prefer to work visually and via a web application that doesn’t require installing software on one’s own device. We believe the duality of having dropR as a Shiny app and dropR as a regular package will allow easier access to solid statistical analyses for all members of the scientific community and help to remove hurdles that may keep researchers from applying dropout analysis. The proliferation of Internet-based research with its higher likelihood of dropout creates the need to more frequently attend to it.

## Data Availability

See next section and official dropR CRAN page for any materials: https://CRAN.R-project.org/package=dropR.

## Footnotes

1 Historically, all web applications were called “web services”. Nowadays, one distinguishes between smaller services called web applications or “web apps” and more comprehensive web services with multiple functions.

## Code availability

All open code is available on GitHub from the iScience Konstanz page at https://github.com/iscience-kn/dropR.

## Declarations

### Open Practices

Materials and all source code are available at https://github.com/iscience-kn/dropR.

### Author Note

This research was first presented at the 4th conference of the European Survey Research Association (ESRA), July 18–22, 2011, Lausanne, Switzerland, in the symposium “Internet-based research: New horizons”, at the 44th annual meeting of the Society for Research in Psychology (SCiP), Long Beach, November 20, 2014, and at the 7th Webdatanet conference, Salamanca, Spain, May 27, 2015. The dropR package was also presented at the user! Conference in Salzburg, Austria, July 9, 2024. Correspondence concerning this article should be addressed to Ulf-Dietrich Reips, reips@uni-konstanz.de, University of Konstanz, Department of Psychology, Box 31, 78457 Konstanz, Germany.
